# P345: Prison health environment and patients safety in Cote d'Ivoire

**DOI:** 10.1186/2047-2994-2-S1-P345

**Published:** 2013-06-20

**Authors:** S Anongba, MF2 Adeoti

**Affiliations:** 1 Health Ministry, Abidjan, Côte d'Ivoire

## Introduction

In Côte d'Ivoire, the prison is a place of social precariousness. It clearly raises the issue of the health of persons under the hand of justice.

## Objectives

Conduct an inventory of the prison health.

## Methods

Cross-sectional study conducted in nine prisons in the country in April 2009 using a survey form filled from interview and documentary collection.

## Results

The prison population is young, predominantly male (95.8%). average rate of overcrowding in the order of 300% of capacity. Approximately 15% of prisoners are drug addicts, 30% consume substantial quantities of alcohol and 80% are smoking more than one pack per day. HIV prevalence among prisoners is higher (4.7%) than the national average (3%). Prevalence is as high as 14.3% among women. Tuberculosis is observed three times more frequently in prisons. Prisons are experiencing serious problems of hygiene leading to recurring epidemics of cholera, typhoid fever, skin disorders. The nutritional situation is characterized by an inmate resulting in power situations often aggravated malnutrition (diarrhea, beriberi) and death in certain establishments (Daloa, Bouaflé, Toumodi, Soubré).

On the level of the infrastructure and equipment, rooms serves as infirmary except for the Prison and of correction of Abidjan where there exists a health centre and the equipment is insufficient or non-existent. 78% of the prisons have a functional lookup table. Only the MACA presents a room of bandage. On the level of human resources: Only the MACA has a staff residing with 1 doctor for 239 prisoners. In the prisons of the interior of the country, 71% of staff is temporary with 1 doctor for 417 and 1 nurse for 454. Moreover, the equipment in drugs is not adapted and irregular and there is an absence of collaboration between the health authorities and the managers of prison, of control of the ministries in charge and budgetary heading for the medical assumption of responsibility for the specialized interventions.

## Conclusion

Health in prison is characterized by a deficit of staff, exceeded prison infrastructures, a lack of means financial and an absence of supervision.

## Disclosure of interest

None declared.

